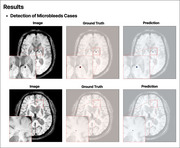# AI‐based Microbleed Detection for ARIA Radiographic Severity Assessment

**DOI:** 10.1002/alz70856_100169

**Published:** 2025-12-24

**Authors:** Saehyun Kim, Wooseok Jung, Seung Hyun Lee, Chong Hyun Suh

**Affiliations:** ^1^ VUNO Inc., Seoul, Seoul, Korea, Republic of (South); ^2^ VUNO Inc., Seocho‐gu, Seoul, Korea, Republic of (South); ^3^ Asan Medical Center, University of Ulsan College of Medicine, Seoul, Korea, Republic of (South)

## Abstract

**Background:**

Microbleed detection is significant in anti‐amyloid therapy (AAT) monitoring to evaluate amyloid‐related imaging abnormalities (ARIA) severity (mild: ≤4, moderate: 5‐9, severe: ≥10), which directly affects treatment decisions. Although T2*/GRE is recommended for the primary diagnostic imaging sequence of ARIA‐H monitoring, the susceptibility‐weighted imaging (SWI) sequence is known to be more sensitive to microbleeds. However, manual assessments are time‐consuming and prone to reader variability. Deep learning‐based automated detection systems can improve the efficiency and reliability of ARIA‐H evaluations.

**Method:**

565 SWI MRI scans (2mm slice thickness) from Asan Medical Center were analyzed, comprising 429 positive and 136 negative cases. The mean age of the cohort was 71.9 ± 10.2 years (202 males, 363 females). A neuroradiologist with 14 years’ experience labeled microbleeds that were defined as hypointense lesions ranging from 2 to 10mm in diameter on SWI. The dataset was split into training, validation, and test subsets at a 3:1:1 ratio. An Attention U‐Net architecture with deep supervision was employed to handle the small size and morphological similarity of cerebral microbleeds. Model validation was performed using the Dice coefficient and the lesion‐level Matthews correlation coefficient (MCC).

**Result:**

A total of 114 test scans were evaluated (86 positive, containing 158 microbleeds, and 28 negative) using a 3 mm lesion center proximity threshold. The model detected 146 of 158 microbleeds, achieving an AUC of 0.872 (sensitivity=0.677, specificity=0.893). False‐positive analysis revealed 103 occurrences in positive scans and 18 in negative scans. Patient‐level metrics included 1.28 microbleeds per scan (95% CI: 1.02–1.54) and 1.06 false positives per scan (95% CI: 0.79–1.37), producing a minimal impact on the ARIA‐H radiological severity classification standard (mild: ≤4 microbleeds, moderate: 5–9, severe: ≥10).

**Conclusion:**

This study presents a robust automatic microbleed detection approach using SWI, facilitating ARIA‐H assessment in diagnosis and severity categorization. Future work will involve multi‐center external validation, supporting additional sequences, and incorporating additional ARIA‐related factors for more comprehensive detection.